# The older prisoner health and social care assessment and plan (OHSCAP) versus treatment as usual: a randomised controlled trial

**DOI:** 10.1186/s12889-021-11965-5

**Published:** 2021-11-10

**Authors:** Katrina Forsyth, Roger T. Webb, Laura Archer Power, Richard Emsley, Jane Senior, Alistair Burns, David Challis, Adrian Hayes, Rachel Meacock, Elizabeth Walsh, Stuart Ware, Jenny Shaw

**Affiliations:** 1 The University of Manchester, Oxford Road, Manchester, M13 9PL UK; 2 Lancashire Police, Saunders Lane, Hutton, PR4 5SB UK; 3 King’s College, London Strand, London, WC2R 2LS UK; 4 Institute of Mental Health, Innovation Park, Triumph Road, Nottingham, NG7 2TU UK; 5 Avon and Wiltshire Mental Health Partnership NHS Trust, Bath NHS House, Newbridge Hill, Bath, BA1 3QE UK; 6 Care UK, Hawker House, 5-6 Napier Court, Napier Road, Reading, Berkshire, RG1 8BW UK; 7 Restore Support Network, Exeter CVS, Wat Tyler House, King William Street, Exeter, EX4 6PD UK

**Keywords:** Old, Prison, Health, Social care, Assessment, Care planning

## Abstract

**Background:**

Older people are the fastest-growing demographic group among prisoners in England and Wales and they have complex health and social care needs. Their care is frequently ad hoc and uncoordinated. No previous research has explored how to identify and appropriately address the needs of older adults in prison. We hypothesised that the Older prisoner Health and Social Care Assessment and Plan (OHSCAP) would significantly increase the proportion of met health and social care needs 3 months after prison entry, compared to treatment as usual (TAU).

**Methods:**

The study was a parallel randomised controlled trial (RCT) recruiting male prisoners aged 50 and over from 10 prisons in northern England. Participants received the OHSCAP or TAU. A clinical trials unit used minimisation with a random element as the allocation procedure. Data analysis was conducted blind to allocation status. The intervention group had their needs assessed using the OHSCAP tool and care plans were devised; processes that lasted approximately 30 min in total per prisoner. TAU included the standard prison health assessment and care. The intention to treat principle was followed. The trial was registered with the UK Clinical Research Network Portfolio (ISRCTN ID: 11841493) and was closed on 30 November 2016.

**Results:**

Data were collected between 28 January 2014 and 06 April 2016. Two hundred and forty nine older prisoners were assigned TAU of which 32 transferred prison; 12 were released; 2 withdrew and 1 was deemed unsafe to interview. Two hundred and fifty three 3 prisoners were assigned the OHSCAP of which 33 transferred prison; 11 were released; 6 withdrew and 1 was deemed unsafe to interview. Consequently, data from 202 participants were analysed in each of the two groups. There were no significant differences in the number of unmet needs as measured by the Camberwell Assessment of Needs – Forensic Short Version (CANFOR-S). The mean number of unmet needs for the OHSCAP group at follow-up was 2.03 (SD = 2.07) and 2.06 (SD = 2.11) for the TAU group (mean difference = 0.088; 95% CI − 0.276 to 0.449, *p* = 0.621).

No adverse events were reported.

**Conclusion:**

The OHSCAP was fundamentally not implemented as planned, partly due to the national prison staffing crisis that ensued during the study period. Therefore, those receiving the OHSCAP did not experience improved outcomes compared to those who received TAU.

**Trial registration:**

Current Controlled Trials: ISRCTN11841493, 25/10/2012.

**Supplementary Information:**

The online version contains supplementary material available at 10.1186/s12889-021-11965-5.

## Background

The number of incarcerated older adults across developed countries has increased markedly in recent times; for example, 17 % of the prison population in England and Wales are now aged 50 and over (13,890). This includes 3970 individuals aged 60 plus; triple the number 15 years age [20]. Older adults in prison have multi-faceted health problems. They have more complex health needs than their peers in the community and younger individuals in prison [6, 7]. It is estimated that between 85 and 93% have some form of physical illness [6, 7, 13], including most commonly, respiratory (8–78%); musculoskeletal (23–57%) and cardiovascular diseases (18–49%) [6, 7, 13, 18, 24]. The most frequent mental illnesses include personality disorder (20–30%); depressive disorder (12–14%) and substance misuse (5%) [6, 7, 12, 14, 18] ([6, 7]; [13, 18]) ([6, 7]; [13, 18]). Older prisoners often have complex social care needs and prisons are ill-equipped to manage these [15]. Hayes et al. [13] found that 11% of their sample of older prisoners had personal care needs and over a third of these were unmet. Older adults residing in prison experience intense anxieties about release and they typically perceive their release planning to be non-existent [8].

There is no national strategy for the care of incarcerated older adults in England and Wales, in spite of numerous calls for one to be developed [1, 5, 12, 14, 23]. Service provision is consequently ad hoc and uncoordinated with targets set out in the National Standards Framework for older people remaining largely unmet in prisons [15]. Department of Health guidance recommends that older adults’ health and social care needs should be assessed, using a specialised assessment on entry into prison. However, only 19% of prisons in England and Wales have introduced such an assessment and these assessments have not been formally evaluated [24]. Furthermore, there are few examples of specialised services/initiatives to provide for the needs of older prisoners and their effectiveness has not been formally evaluated [24]. It is important that the health and social care needs of older prisoners are adequately met. It has been claimed that current service provision in England and Wales is sub-standard and even unlawful due to its failure to adhere to the Disability Discrimination Act, Equality Act and article 8 of the European Convention on Human Rights [13]. Furthermore, most prisoners will be released at some point in time. Prison is a unique time to engage individuals with services and meet needs. Adequately meeting older prisoners’ needs could lead to preventive strategies that reduce costs when prisoners are released and may also reduce the likelihood of reoffending after release. Consequently, the failure to meet older adults’ health and social care needs when they are residing in prison has a long-term detrimental impact.

Previous studies have explored the current situation and identified difficulties for older adults in prison, There have been no previous research evaluating health and social care initiatives for older adults in prison.. This is the first study to evaluate an intervention for this group. The Older prisoner Health and Social Care Assessment and Plan (OHSCAP) was developed through ‘action learning’ as part of a previous study [24]. It is a structured approach to identifying and addressing older prisoners’ health and social care needs. Findings from the pilot study suggested that the OHSCAP allowed quick resolution for day-to-day prison based problems; early referral to health and support organisations and better multi-agency working [24]. The aim of the current study was to evaluate the effectiveness and acceptability of the OHSCAP in comparison to Treatment as Usual (TAU).

## Methods

### Inclusion/exclusion criteria

To be eligible for inclusion, participants were male, aged 50 or over and with a known release date (convicted) or likely release date (unconvicted) of at least 3 months after their prison entry date. The following groups of were excluded: i) those who did not have the capacity to consent; ii) those deemed by prison or healthcare staff as being unsafe to interview alone due to their current risk assessment; and iii) those previously included in the study. Written informed consent was obtained from all participants.

### Study design

The study was designed to evaluate the OHSCAP. It consisted of a parallel two group RCT with 1:1 individual participant allocation to either the OHSCAP intervention plus TAU (intervention group) or TAU alone (control group). The study aimed to recruit older male prisoners aged 50 and over from ten prisons in England including open, training, and high secure establishments. Participants were allocated to either the OHSCAP intervention plus TAU (intervention group) or TAU alone (control group). Randomisation was undertaken by the Manchester Academic Health Science Centre Clinical Trials Unit (MAHSC CTU). The MAHSC CTU provided a telephone-based central randomisation service for the trial. The allocation method was minimisation with a random element using imbalance scores over the margins of two factors: Institution and baseline number of unmet needs (0, 1, 2, 3, 4+).

### The OHSCAP intervention and its delivery

The OHSCAP was developed and implemented as part of a previous study funded by the National Institute for Health Research (NIHR) Service Delivery and Organisation (SDO) programme. An Action Learning Group (including prisoners, NHS staff and prison staff) at one prison in England developed the OHSCAP [24]. The OHSCAP is a structured approach for identifying and managing the health and social care needs of older adults residing in prison. The previous study suggested that the OHSCAP was acceptable to older people and staff, could be integrated into current prison/healthcare processes, assisted effective multi-agency working, provided an opportunity for older adults to raise their concerns that would have otherwise gone unreported, and could be successfully conducted by a prison officer. The OHSCAP is paper based with information collected and uploaded onto existing prison, health and offender manager computer systems. It consists of an assessment and a care plan as well as reviews of these two components, as follows:

*Assessment*: includes a series of open questions to facilitate discussion and is divided into three key parts, namely: (i) social care; (ii) health and well-being; (iii) release planning. The social assessment includes open questions around relationships, activities, and mobility. The well-being assessment includes exploratory questions around emotional health, physical health, medications, and treatment. A section for ‘other’ concerns is also incorporated. The final section of the assessment includes open questions around discharge planning. A series of ‘trigger’ open questions are included for each of these sections.

*Care plan*: consists of a matrix with the following five columns: 1) Issue raised from assessment; 2) Aim of the proposed action; 3) Action (by whom and when); 4) Date to be reviewed and rationale; 5) Status of action.

*Review section*: includes space for a date and details of: 1) Progress since last review; 2) Action planned; and 3) Next review with rationale.

The assessment was conducted approximately one to 2 weeks after an older prisoner entered prison. The assessor conducted the assessment one-to-one with each older prisoner. The care plan was completed in conjunction with the older prisoner and they were provided with a copy of their OHSCAP. In addition, a summary of the OHSCAP was entered onto the prison computerised information system (C-NOMIS) and a copy of the OHSCAP was scanned onto the prison computerised clinical records (SystmOne) and probation computer records (Offender Assessment System - OASys).

It was initially intended that the Older Prisoner Leads, who are prison officers, would deliver the intervention. However, in 6 of the 10 study sites healthcare workers delivered the OHSCAP, as this was deemed by senior managers at the sites to be more appropriate or more achievable within their prison at the time the project was being set up. This was largely due to the benchmarking process taking place at the time, which resulted in a reduction in prison officers and the loss of some roles, including the Disability Liaison Officer (DLO), who was also responsible for supporting older prisoners.

All of the OHSCAP facilitators were trained to deliver the OHSCAP, in line with the OHSCAP manual (available here: http://www.ohrn.nhs.uk/OHRNResearch/OHSCAP/Manual.pdf). There were further training sessions held at The University of Manchester, which were attended by facilitators from all study sites. Ongoing support was also offered by one of the investigators (EW), who acted as a mentor to facilitators and was contactable by telephone and email should they have any questions or need any reassurance.

Treatment as usual entailed the standard non age-specific health assessment carried out at prison entry [11]. Other support provided within treatment as usual varied between prisons but included older prisoner social groups, peer carers and ‘healthy men’ checks. It has been identified that identification of health and social needs and care planning is generally ad hoc and deemed inadequate [24].

Data were collected at baseline and three-month follow-up. The following assessments were made at baseline: Camberwell assessment of need – Short Forensic Version [27]([primary outcome measure]. The secondary outcome measures were Geriatric Depression Scale – Short Form (GDS-15) [26]; EQ-5D-5L [16]; Bristol Activities of Daily Living Scale [2] and the bespoke OHSCAP research tool designed by the research team for this study, to measure the extent to which specific health and social care needs had been addressed (supplementary material 1). PriSnQuest [25] and the Burvil Grid [4] were also applied at baseline to describe the sample.

### Statistical analysis

Analysis was conducted in Statistical package for the Social Science (SPSS) version 20. Quantitative data analysis was conducted blind to allocation status. From our previous work, the mean number of unmet needs of 100 older prisoners was estimated to be 2.71 (sd = 2.65, range 0–25, median = 2) [24]. To detect a 30% reduction to a mean of 1.90 with 80% power, 196 participants were required in each trial arm at three-month follow-up. Accounting for 15% attrition, we aimed to recruit 504 at baseline. All analyses were carried out according to the intention to treat (ITT) principle. The primary hypothesis for the change in the mean number of unmet needs as measured by the CANFOR-S was analysed using linear regression models. We adjusted for baseline characteristics used in the minimisation process i.e. site and number of unmet needs at baseline. We used bootstrapping to account for the skewness in the outcome of the data. As a sensitivity analysis, we fitted a Poisson model to analyse the data as counts along with a log-linear negative binominal model to assess for over-dispersion.

## Results

### Participants

**Fig. 1:**
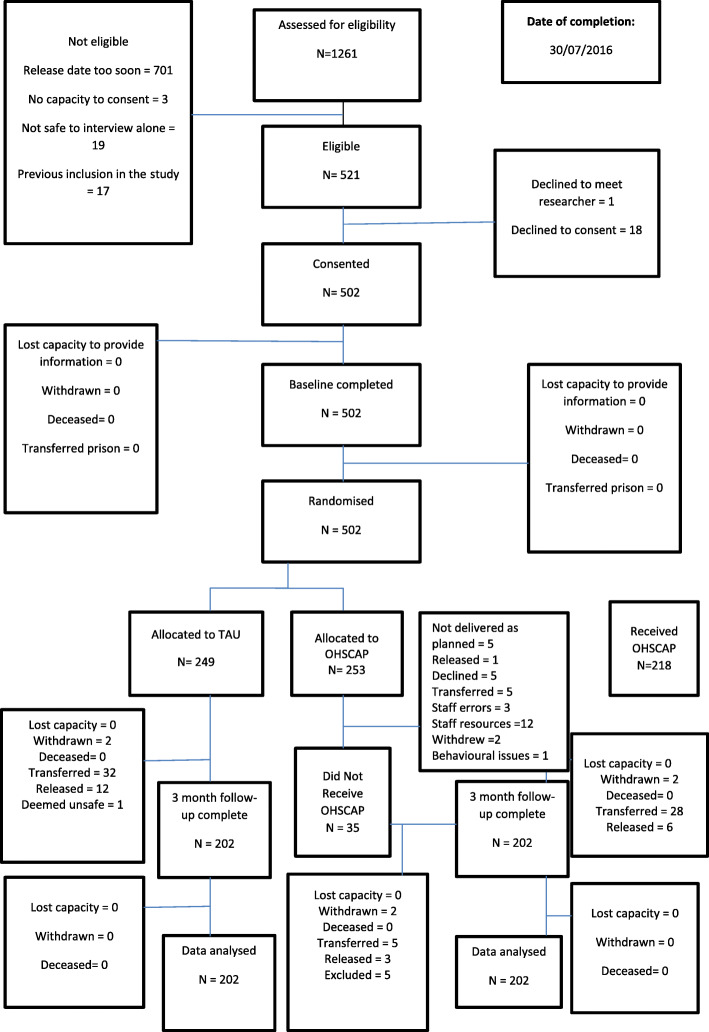
CONSORT diagram

Between January 1, 2014 and April 6th 2016, 502 participants were enrolled from 10 prisons, with 249 allocated to receive TAU and 253 allocated to receive the OHSCAP (Fig. 1). Baseline characteristics were well balanced across treatment groups (Table 1), including their index offence, prisoner status and whether or not they had been in prison before. The majority were of White British ethnicity and were convicted (sentenced) prisoners. They were most commonly convicted of sexual offences, followed by drug and violent offences. The majority scored less than 3 on PriSnQuest (80%) indicating they did not require any further mental health assessment at the time the interview was conducted. The most common mental illness was generalised anxiety disorder (6%, identified via OPCRIT). The mean number of body systems acutely affected according to the Burvil Grid was 0.2 and chronically affected was 2.1.

**Table 1:** Baseline characteristics of the intention-to-treat population

	TAU (***n*** = 249)	OHSCAP (***n*** = 248)
**Age**	59 (7.8)	57 (7.0)
**Ethnic origin**		
White	215 (86%)	231 (93%)
Black	14 (6%)	2 (1%)
Asian	10 (4%)	6 (2%)
other	10 (4%)	9 (4%)
**Index offence**		
Violence	33 (13%)	29 (12%)
Sexual offence	98 (39%)	109 (44%)
Drug offences	52 (21%)	36 (14%)
**Prisoner status**		
Remand	41 (17%)	37 (15%)
Convicted, unsentenced	13 (5%)	8 (3%)
Convicted, sentenced	195 (78%)	203 (82%)
**Been in prison before**		
Yes	132 (53%)	123 (49%)
**OpCriT Diagnosis**		
Psychosis/Schizophrenia	8 (4%)	5 (2%)
Depression	5 (2%)	8 (4%)
Anxiety disorder	17 (8%)	16 (8%)
Personality Disorder	1 (0%)	1 (0%)
Harmful use of drugs	25 (12%)	9 (4%)
Harmful use of alcohol	11 (5%)	15 (7%)
Other	5 (2%)	3 (1%)
**Burvil Grid**		
Chronic Severity	4.3 (3.3)	3.5 (3.2)
Chronic Disability	3.7 (3.4)	2.9 (2.97)

There were no significant differences between the two groups at 3 months follow-up for the primary outcome measure of the total number of unmet needs or any of the individual domains of the CANFOR-S (Table 2). When the log linear negative binominal regression model was run the results were unchanged from the Poisson model, indicating that the Poisson model was not over-dispersed.

**Table 2:** Primary and secondary outcome measure results

	TAU (***n*** = 202)	OHSCAP (***n*** = 202)	Relative risk or mean difference (95% CI)	95% CI
**Primary Outcome**				
Total no. unmet need	2.06 (2.114)	2.03 (2.066)	0.088 ^a^-0.078 ^b^	−0.276 to 0.449 − 2.16 to 0.061
OHSCAP bespoke total	60.15(7.624)	61.83(6.546)	−0.166^a^	− 3.996 to 4.231
GDS scale				
0–5 normal	135 (67%)	142 (70%)	1.033	0.617 to
6–15 depressive symp.	67 (31%)	59 (29%)		1.732

^a^Linear regression with bootstrapping ^b^Poisson model

Thirty one percent scored between 6 and 15 on the GDS indicating depressive symptoms. There were no statistically significant differences between groups (Table 2).

The OHSCAP bespoke Likert scale included the following options: ‘not at all’; ‘very little’; ‘somewhat’ and ‘to a great extent’. For clarity, the mean responses are included in Table 2. Issues that were more likely to be met were access to a GP on release and; collecting meals and showering whilst in prison (mean = 2.95, 2.87 and 2.84 respectively). Problems that were less likely to be met included information about release processes, sleep and boredom (with means of 1.98, 2.06 and 2.06, respectively). There was a statistically significant difference between groups for prisons’ ability to hear instructions from prison officers (O.173, *p* = 0.014, 95% CI - 0.30 to 0.311). There were no other statistically significant differences between groups.

For the TAU group, the mean EQ-5D-5L utility score was 0.833 at baseline and 0.867 at follow-up in the complete-case sample. For the group receiving the OHSCAP, the mean baseline utility was 0.852 and the mean utility at follow-up was 0.866. Over the study period, the mean unadjusted QALY for the TAU group was 0.186 and for the OHSCAP group was 0.187. This indicated that there is no incremental effect of the OHSCAP over and above TAU in the unadjusted figures.

## Discussion

The hypotheses was that the OHSCAP would significantly increase the proportion of met health and social care needs 3 months after prison entry, compared to TAU controls. However, there was no difference in the number of unmet health and social care needs between the TAU and OHSCAP group at the 3 month follow up. There were also no differences between the groups when depression or activities of daily living needs were examined. Specific health and social care need domains were examined separately. There were no differences between groups for how well specific health and social care needs were met, with the exception of hearing instructions. Prisoners who received the OHSCAP were more likely to have their needs met for this domain, than those in the TAU group.

The fundamental reason for the lack of difference between the TAU and OSCAP groups is that the OHSCAP was not implemented as intended [9]. An audit of all available OHSCAPs was conducted (68%) to identify both the fidelity of implementation and the quality of the care planning. The OHSCAP manual stipulated that the assessments should be completed 7 to 14 days after prison entry. However, the audit found that the OHSCAPs were completed on average 20 days after arrival in prison (range 4 to 63). Similarly, care plans should have been produced after each assessment. However, care plans were documented for less than half of the OHSCAPs reviewed (43%). Furthermore, no action was reportedly taken in 43% of cases where problems were identified.

The nested qualitative study provided a useful insight into why the OHSCAPs had not been properly implemented [10]. Semi-structured interviews with 14 prisoners and 11 staff members identified concerns about healthcare and prison ‘silos’ resulting in a lack of meaningful multi-agency and partnership working. Prisoners also reported that they deemed it unacceptable for prison officers to be facilitating the intervention. Most strikingly, staff reported that they were operating within a ‘broken prison system’, because of the recent marked staff reductions. Staff reported that meeting basic needs such as ensuring that all prisoners had opportunities for showers and remained safe was more challenging since the staff reduction, and therefore completing the OHSCAPs was a low priority.

The findings of this study have important policy and practice implications. At the time of data collection, prisons were in crisis. The current study was conceived and in process at a time when the then coalition government introduced policies with the intention of reducing staffing levels across the National Offender Management Service as a whole. Benchmarking involved an attempt to reduce costs across the prison system of England and Wales by decreasing the number of prison officers [17]. These reductions were achieved through alterations to the prison regimes. Between March 2010 and September 2016, grade 3 to 5 operational prison officer numbers fell by 26.3% in public sector prisons, excluding structural changes (prison closures, movement between public/private operation) [19]. Data collection itself was undertaken when the benchmarking process came into operation. This impacted upon the ability of staff to implement the OHSCAP as intended.

The detrimental impacts of these staff shortages were widely described by participants, both professionals and residents, during the qualitative interviews [9]. After data collection for this study was completed, the government White Paper ‘Prison Safety and Reform’ acknowledged serious problems with the prison system and the need for change [19]. The paper proposed several changes to the prison system, including increasing staff-to-prisoner ratios through the recruitment of an additional 2500 prison officers. By December 2017, prison officer numbers were at the highest number they have been since September 2013, increasing by 161% between December 2016 and December 2017 [20]. However, the newly recruited prison officers lacked the experience that previous staff held [3].

The House of Commons Justice Select Committee has stipulated that:

“The key explanatory factor for the obvious deterioration in standards over the last year is that a significant number of prisons have been operating at staffing levels below what is necessary to maintain reasonable, safe and rehabilitative regimes.” [17].

This loss of prison officer numbers has been linked, by a range of media, political and societal informants to a range of complex and inter-related negative outcomes with significant increases in self-inflicted deaths, self-harm incidents and violence from 2015 to 2016. More recently, there has been a welcome fall in self-inflicted deaths, but self-harm and violence towards both prisoners and staff continue to rise. It is hoped that the roll out of the new Offender Management in Custody (OMiC) model will have a positive impact. This aims to increase staffing levels on residential units and provide an officer ‘key worker’ for every prisoner. However, there has been a significant increase in resignations amongst prison officers in recent years [3] and it should be acknowledged that the recruitment of a relatively small (in comparison with the overall reduction in the prison officer workforce since 2010) number of new recruits will, arguably, not compensate for the prison officers with considerable experience who have been lost since 2013. Consequently, the reduction in prison offers needs addressing in policy in order to increase safety standards and improve the likelihood of important health and social care initiatives being fully imbedded into the prison system.

Alternatives to increasing prison officer numbers to improve standards of care may also be required. To better reflect equivalency with the community and to improve the quantity, scope and targeting of services further research should explore and identify the role other prisoners and third sector organisations (such as older adult specialist services) could play in identifying and appropriately addressing older prisoners’ health and social care needs. There have been recent moves towards using prisoner peers to address older prisoner health and social care needs, but the appropriateness and effectiveness of such interventions is largely unknown [28].

The introduction of the Care Act (2014) also occurred during the data collection phase of this study. Fundamentally, this meant that local authorities became responsible for the provision of social care for prisoners [22]. The OHSCAP was designed to complement the Care Act by providing a system for meeting the social care needs of older prisoners who did not meet the high threshold for social care packages set by local authorities. Early research suggests that many different models of social care have been adopted by local authorities with varying degrees of success [28]. This marked change in social care provision may have further impeded the successful implementation of the OHSCAP at a time when there was confusion over the provision of social care in prisons and significant change. Further research is required to understand how effective models of social care can be developed and implemented in prison.

Although the OHSCAP was not delivered as intended, it is important to discuss the impact of potential limitations of the study. The possibility of contamination between the TAU and treatment arm was carefully considered. An individual-level randomised design was selected, and consequently individuals within the same prison were receiving both TAU and OHSCAP, because it was anticipated that there would be minimal contamination between the two groups. This was thought be the case because older prisoners were not systematically identified on entry into prison within the TAU arm and therefore the Older Prisoner Lead does not usually come into contact with these older prisoners.

The research tools selected were the most appropriate ones that were available; however they had some limitations. A number of participants indicated that many of the discrete domain items in the CANFOR-S were not applicable to their current situation in prison, or at all, given their age, (for example asking about needs in relation to childcare responsibilities). In addition, the CANFOR-S considered needs to either be met or unmet, but it is unlikely that some health and social care needs are ever fully met as they are ongoing and changeable in their nature and / or severity. Findings from our previous research suggest that older adults in prison are less likely to raise concerns than their younger counterparts [24]; consequently participants in this study may not have always disclosed if they were experiencing unmet needs. In spite of the limitations of the CANFOR-S this tool was considered by the authors to be the best one available for measuring health and social care needs within the prison setting and has been successfully used with this population in previous studies [13, 24]. It was decided that a three-month follow-up period should allow sufficient time for initial needs to be met. The CANFOR measures whether or not prisoners are receiving some beneficial assistance. The research team perceived 3 months to be sufficient time for the prisoners to begin to get suitable help, and we also wished to minimise attrition.

There were also some limitations of the tools used to measure the secondary outcomes. The GDS-15 was not designed for use in prison. However, the scale has been used with older prisoners in a previous study. In that research, one question was adapted from ‘do you prefer to stay at home rather than go out and do new things?’ to ‘do you go ‘on association’?’ [21]. ‘On association’ is the term used describe those residing in prison leaving their cell and socialising with other residents. We adopted the same approach. Additionally, very few activities of daily living needs were identified using the BADL (Table 3, supplementary Table 2). This tool has been used in previous older prisoner studies [13]. However, it is designed for use with dementia patients and is perhaps not sensitive enough to identify activities of daily living needs among either older adults not experiencing dementia, and among those living in a limiting institutional setting.

We acknowledged at the planning stages that participants would unavoidably became aware of which group they have been allocated to when they received the intervention. Furthermore, the researchers knew which group some of the participants belonged to because 14 of the participants in the intervention group were invited to take part in qualitative semi-structured interviews.

The CANFOR-S was used because, it was the most appropriate available tool for assessing unmet health and social care needs within the prison population. The research team were, however, aware that there were certain domains of the CANFOR-S that the OHSCAP specifically aimed to address and some domains of the CANFOR-S that the OHSCAP did not aim to address. The research team therefore felt it would be useful to analyse the data separately for the specific domains of the CANFOR-S that were considered most relevant to the OHSCAP. The aim of this analysis was to gain a more detailed understanding of the specific domains of the CANFOR-S that the OHSCAP appeared to assist more with and which domains the OHSCAP was less able to address.

Data collection targets were closely monitored throughout the study. Three months after the trial was initiated it became apparent that we needed to recruit additional sites to meet the follow-up target of 392. Additional sites were selected for pragmatic reasons whilst still ensuring a range of prison types were included. The number of sites consequently increased from four to ten. This change was made in consultation with the Clinical Trials Unit (CTU). While monitoring the trial’s progression it became apparent that its attrition rate (of almost 20%) was much higher than the expected 10%. This was mainly due to retention issues in the local (remand) prison sites, where it proved harder than expected to identify individuals who would remain in custody for the three-month follow-up period. As a result of this, after the initial 6 months of data collection, we extended our recruitment period and increased our baseline recruitment target to a maximum of 502 participants at baseline, from the original target of 462. This change was made in conjunction with the CTU.

## Conclusion

No significant differences, in terms of met health and social care needs, were observed between the OHSCAP and TAU groups. However, the OHSCAP was not delivered as intended, largely due to prisons experiencing a staffing crisis. Since the study was conducted, the number of prison offers has been increased, but many experienced officers have been lost to the service. This study has highlighted the challenge of conducting RCTs in a significantly under-resourced and deteriorating prison environment. Prisons require stable experienced staffing for health and social care interventions to be successful. Future interventions and research studies should take measures to minimise the impact of policy and staffing changes, where possible. This may include the use of peer carers, the third sector and thorough and inclusive implementation strategies.

## Supplementary Information

**Additional file 1: Supplementary Material 1.** OHSCAP bespoke questionnaire, Designed by the research team to capture any differences in specific prison based activities of daily living

**Additional file 2: Supplementary Material 2.** Table 3, Functional health and well-being as measured by the Bristol Activities of Daily Living Scale at 3 months follow-up

## Data Availability

The datasets used and/or analysed during the current study are available from the corresponding author on reasonable request.

## Abbreviations

HMPPS: Her Majesty’s Prison and Probation Service
OHSCAP: Older prisoner Health and Social Care Assessment and Plan
TAU: Treatment as Usual
