# Supplementary material for: The older prisoner health and social care assessment and plan (OHSCAP) versus treatment as usual: a randomised controlled trial
Source: BMC Public Health. 2021 Nov 10;21:2061. doi: 10.1186/s12889-021-11965-5 (PMC8579542; doi:10.1186/s12889-021-11965-5)
Supplement: Supplementary file 1 — Additional file 1: Supplementary Material 1. OHSCAP bespoke questionnaire, Designed by the research team to capture any differences in specific prison based activities of daily living [file 12889_2021_11965_MOESM1_ESM.docx]

| **Supplementary material 1: OHSCAP bespoke tool, Q1** |  |  |
| --- | --- | --- |

|  | | | | | | | | | | | | | |
| --- | --- | --- | --- | --- | --- | --- | --- | --- | --- | --- | --- | --- | --- |
| To what extent are you currently experiencing difficulties in the following areas? | | | | | | | | | | | | | |
|  | | | | | | | | | | | | | |
|  |  | | |  | | **NOT AT ALL** | **VERY LITTLE** | **SOMEWHAT** | **TO A GREAT EXTENT** | **N/A** | **NOT STATED** |  | |
| **1 SOCIAL** | | | | | | | | | | | | | |
|  | | | | | | | | | | | | | |
|  | 1.1 | | Relationships | | |  |  |  |  |  |  |  | |
|  | | 1.1.1 | | | Telephoning family/friends | 00 | 01 | 02 | 03 | 88 | 98 |  | |
|  | | 1.1.2 | | | Receiving visits from family/friends | 00 | 01 | 02 | 03 | 88 | 98 |  | |
|  | | 1.1.3 | | | Giving/receiving letters to/from family/friends | 00 | 01 | 02 | 03 | 88 | 98 |  | |
|  | | 1.1.3 | | | Bullying by other prisoners | 00 | 01 | 02 | 03 | 88 | 98 |  | |
|  | | 1.1.4 | | | Mixing/socialising with other prisoners | 00 | 01 | 02 | 03 | 88 | 98 |  | |
|  | | | | | | | | | | | | | |
|  |  | | | | | | | | | | | |  |
|  | 1.2 | | Activities | | |  |  |  |  |  |  |  | |
|  | | 1.2.1 | | | Boredom | 00 | 01 | 02 | 03 | 88 | 98 |  | |
|  | | 1.2.2 | | | Lack of appropriate education | 00 | 01 | 02 | 03 | 88 | 98 |  | |
|  | | 1.2.3 | | | Lack of appropriate employment | 00 | 01 | 02 | 03 | 88 | 98 |  | |
|  | | | | | | | | | | | | | |
|  |  | |  | | |  |  |  |  |  |  | |  |
|  | 1.3 | | Mobility | | |  |  |  |  |  |  |  | |
|  | | 1.3.1 | | | Accessing parts of the prisons | 00 | 01 | 02 | 03 | 88 | 98 |  | |
|  | | 1.3.2 | | | Collecting meals | 00 | 01 | 02 | 03 | 88 | 98 |  | |
|  | | 1.3.3 | | | Getting in and out of bed | 00 | 01 | 02 | 03 | 88 | 98 |  | |
|  | | 1.3.4 | | | Showering/washing | 00 | 01 | 02 | 03 | 88 | 98 |  | |
|  | | | | | | | | | | | | | |
| **2 WELLBEING** | | | | | | | | | | | | | |
|  | | | | | | | | | | | | | |
|  | 2.1 | | Emotional | | |  |  |  |  |  |  |  | |
|  | | 2.1.1 | | | Feeling safe | 00 | 01 | 02 | 03 | 88 | 98 |  | |
|  | | 2.1.2 | | | Sleep | 00 | 01 | 02 | 03 | 88 | 98 |  | |
|  | | 2.1.3 | | | Stress | 00 | 01 | 02 | 03 | 88 | 98 |  | |
|  | | | | | | | | | | | | | |
|  |  | |  | | |  |  |  |  |  |  | |  |
|  | 2.2 | | Physical | | |  |  |  |  |  |  |  | |
|  | | 2.2.1 | | | Glasses/Contact lenses | 00 | 01 | 02 | 03 | 88 | 98 |  | |
|  | | 2.2.2 | | | Hearing instructions | 00 | 01 | 02 | 03 | 88 | 98 |  | |
|  | | | | | | | | | | | | | |
|  |  | |  | | |  |  |  |  |  |  | |  |
|  | 2.3 | | Medications and treatment | | |  |  |  |  |  |  |  | |
|  | | 2.3.1 | | | Delays in receiving medication | 00 | 01 | 02 | 03 | 88 | 98 |  | |
|  | | 2.3.2 | | | Receiving appropriate medication | 00 | 01 | 02 | 03 | 88 | 98 |  | |
|  | | | | | | | | | | | | | |
| **3 DISCHARGE FROM PRISON** | | | | | | | | | | | | | |
|  | | | | | | | | | | | | | |
|  | 3.1 | | Finances | | | 00 | 01 | 02 | 03 | 88 | 98 |  | |
|  | | | | | | | | | | | | | |
|  | 3.2 | | Accommodation | | | 00 | 01 | 02 | 03 | 88 | 98 |  | |
|  | | | | | | | | | | | | | |
|  | 3.3 | | Information about release processes | | | 00 | 01 | 02 | 03 | 88 | 98 |  | |
|  | | | | | | | | | | | | | |
|  | 3.4 | | Access to GP in the community | | | 00 | 01 | 02 | 03 | 88 | 98 |  | |
|  | | | | | | | | | | | | | |
|  | | | | | | | | | | | | | |

| **OHSCAP bespoke tool, Q2** |  |  |
| --- | --- | --- |

|  | | | | | | | | | | | | | |
| --- | --- | --- | --- | --- | --- | --- | --- | --- | --- | --- | --- | --- | --- |
| To what extent have you received help in the following areas? | | | | | | | | | | | | | |
|  | | | | | | | | | | | | | |
|  |  | | |  | | **NOT AT ALL** | **VERY LITTLE** | **SOMEWHAT** | **TO A GREAT EXTENT** | **N/A** | **NOT STATED** |  | |
| **1 SOCIAL** | | | | | | | | | | | | | |
|  | | | | | | | | | | | | | |
|  | 1.1 | | Relationships | | |  |  |  |  |  |  |  | |
|  | | 1.1.1 | | | Telephoning family/friends | 00 | 01 | 02 | 03 | 88 | 98 |  | |
|  | | 1.1.2 | | | Receiving visits from family/friends | 00 | 01 | 02 | 03 | 88 | 98 |  | |
|  | | 1.1.3 | | | Giving/receiving letters to/from family/friends | 00 | 01 | 02 | 03 | 88 | 98 |  | |
|  | | 1.1.3 | | | Bullying by other prisoners | 00 | 01 | 02 | 03 | 88 | 98 |  | |
|  | | 1.1.4 | | | Mixing/socialising with other prisoners | 00 | 01 | 02 | 03 | 88 | 98 |  | |
|  | | | | | | | | | | | | | |
|  |  | | | | | | | | | | | |  |
|  | 1.2 | | Activities | | |  |  |  |  |  |  |  | |
|  | | 1.2.1 | | | Boredom | 00 | 01 | 02 | 03 | 88 | 98 |  | |
|  | | 1.2.2 | | | Lack of appropriate education | 00 | 01 | 02 | 03 | 88 | 98 |  | |
|  | | 1.2.3 | | | Lack of appropriate employment | 00 | 01 | 02 | 03 | 88 | 98 |  | |
|  | | | | | | | | | | | | | |
|  |  | |  | | |  |  |  |  |  |  | |  |
|  | 1.3 | | Mobility | | |  |  |  |  |  |  |  | |
|  | | 1.3.1 | | | Accessing parts of the prisons | 00 | 01 | 02 | 03 | 88 | 98 |  | |
|  | | 1.3.2 | | | Collecting meals | 00 | 01 | 02 | 03 | 88 | 98 |  | |
|  | | 1.3.3 | | | Getting in and out of bed | 00 | 01 | 02 | 03 | 88 | 98 |  | |
|  | | 1.3.4 | | | Showering/washing | 00 | 01 | 02 | 03 | 88 | 98 |  | |
|  | | | | | | | | | | | | | |
| **2 WELLBEING** | | | | | | | | | | | | | |
|  | | | | | | | | | | | | | |
|  | 2.1 | | Emotional | | |  |  |  |  |  |  |  | |
|  | | 2.1.1 | | | Feeling safe | 00 | 01 | 02 | 03 | 88 | 98 |  | |
|  | | 2.1.2 | | | Sleep | 00 | 01 | 02 | 03 | 88 | 98 |  | |
|  | | 2.1.3 | | | Stress | 00 | 01 | 02 | 03 | 88 | 98 |  | |
|  | | | | | | | | | | | | | |
|  |  | |  | | |  |  |  |  |  |  | |  |
|  | 2.2 | | Physical | | |  |  |  |  |  |  |  | |
|  | | 2.2.1 | | | Glasses/Contact lenses | 00 | 01 | 02 | 03 | 88 | 98 |  | |
|  | | 2.2.2 | | | Hearing instructions | 00 | 01 | 02 | 03 | 88 | 98 |  | |
|  | | | | | | | | | | | | | |
|  |  | |  | | |  |  |  |  |  |  | |  |
|  | 2.3 | | Medications and treatment | | |  |  |  |  |  |  |  | |
|  | | 2.3.1 | | | Delays in receiving medication | 00 | 01 | 02 | 03 | 88 | 98 |  | |
|  | | 2.3.2 | | | Receiving appropriate medication | 00 | 01 | 02 | 03 | 88 | 98 |  | |
|  | | | | | | | | | | | | | |
| **3 DISCHARGE FROM PRISON** | | | | | | | | | | | | | |
|  | | | | | | | | | | | | | |
|  | 3.1 | | Finances | | | 00 | 01 | 02 | 03 | 88 | 98 |  | |
|  | | | | | | | | | | | | | |
|  | 3.2 | | Accommodation | | | 00 | 01 | 02 | 03 | 88 | 98 |  | |
|  | | | | | | | | | | | | | |
|  | 3.3 | | Information about release processes | | | 00 | 01 | 02 | 03 | 88 | 98 |  | |
|  | | | | | | | | | | | | | |
|  | 3.4 | | Access to GP in the community | | | 00 | 01 | 02 | 03 | 88 | 98 |  | |
|  | | | | | | | | | | | | | |
|  | | | | | | | | | | | | | |

| **OHSCAP bespoke tool, Q3** |  |  |
| --- | --- | --- |

|  | | | | | | | | | | | | | |
| --- | --- | --- | --- | --- | --- | --- | --- | --- | --- | --- | --- | --- | --- |
| To what extent are your current needs being met in the following areas? | | | | | | | | | | | | | |
|  | | | | | | | | | | | | | |
|  |  | | |  | | **NOT AT ALL** | **VERY LITTLE** | **SOMEWHAT** | **TO A GREAT EXTENT** | **N/A** | **NOT STATED** |  | |
| **1 SOCIAL** | | | | | | | | | | | | | |
|  | | | | | | | | | | | | | |
|  | 1.1 | | Relationships | | |  |  |  |  |  |  |  | |
|  | | 1.1.1 | | | Telephoning family/friends | 00 | 01 | 02 | 03 | 88 | 98 |  | |
|  | | 1.1.2 | | | Receiving visits from family/friends | 00 | 01 | 02 | 03 | 88 | 98 |  | |
|  | | 1.1.3 | | | Giving/receiving letters to/from family/friends | 00 | 01 | 02 | 03 | 88 | 98 |  | |
|  | | 1.1.3 | | | Bullying by other prisoners | 00 | 01 | 02 | 03 | 88 | 98 |  | |
|  | | 1.1.4 | | | Mixing/socialising with other prisoners | 00 | 01 | 02 | 03 | 88 | 98 |  | |
|  | | | | | | | | | | | | | |
|  |  | | | | | | | | | | | |  |
|  | 1.2 | | Activities | | |  |  |  |  |  |  |  | |
|  | | 1.2.1 | | | Boredom | 00 | 01 | 02 | 03 | 88 | 98 |  | |
|  | | 1.2.2 | | | Lack of appropriate education | 00 | 01 | 02 | 03 | 88 | 98 |  | |
|  | | 1.2.3 | | | Lack of appropriate employment | 00 | 01 | 02 | 03 | 88 | 98 |  | |
|  | | | | | | | | | | | | | |
|  |  | |  | | |  |  |  |  |  |  | |  |
|  | 1.3 | | Mobility | | |  |  |  |  |  |  |  | |
|  | | 1.3.1 | | | Accessing parts of the prisons | 00 | 01 | 02 | 03 | 88 | 98 |  | |
|  | | 1.3.2 | | | Collecting meals | 00 | 01 | 02 | 03 | 88 | 98 |  | |
|  | | 1.3.3 | | | Getting in and out of bed | 00 | 01 | 02 | 03 | 88 | 98 |  | |
|  | | 1.3.4 | | | Showering/washing | 00 | 01 | 02 | 03 | 88 | 98 |  | |
|  | | | | | | | | | | | | | |
| **2 WELLBEING** | | | | | | | | | | | | | |
|  | | | | | | | | | | | | | |
|  | 2.1 | | Emotional | | |  |  |  |  |  |  |  | |
|  | | 2.1.1 | | | Feeling safe | 00 | 01 | 02 | 03 | 88 | 98 |  | |
|  | | 2.1.2 | | | Sleep | 00 | 01 | 02 | 03 | 88 | 98 |  | |
|  | | 2.1.3 | | | Stress | 00 | 01 | 02 | 03 | 88 | 98 |  | |
|  | | | | | | | | | | | | | |
|  |  | |  | | |  |  |  |  |  |  | |  |
|  | 2.2 | | Physical | | |  |  |  |  |  |  |  | |
|  | | 2.2.1 | | | Glasses/Contact lenses | 00 | 01 | 02 | 03 | 88 | 98 |  | |
|  | | 2.2.2 | | | Hearing instructions | 00 | 01 | 02 | 03 | 88 | 98 |  | |
|  | | | | | | | | | | | | | |
|  |  | |  | | |  |  |  |  |  |  | |  |
|  | 2.3 | | Medications and treatment | | |  |  |  |  |  |  |  | |
|  | | 2.3.1 | | | Delays in receiving medication | 00 | 01 | 02 | 03 | 88 | 98 |  | |
|  | | 2.3.2 | | | Receiving appropriate medication | 00 | 01 | 02 | 03 | 88 | 98 |  | |
|  | | | | | | | | | | | | | |
| **3 DISCHARGE FROM PRISON** | | | | | | | | | | | | | |
|  | | | | | | | | | | | | | |
|  | 3.1 | | Finances | | | 00 | 01 | 02 | 03 | 88 | 98 |  | |
|  | | | | | | | | | | | | | |
|  | 3.2 | | Accommodation | | | 00 | 01 | 02 | 03 | 88 | 98 |  | |
|  | | | | | | | | | | | | | |
|  | 3.3 | | Information about release processes | | | 00 | 01 | 02 | 03 | 88 | 98 |  | |
|  | | | | | | | | | | | | | |
|  | 3.4 | | Access to GP in the community | | | 00 | 01 | 02 | 03 | 88 | 98 |  | |
|  | | | | | | | | | | | | | |
|  | | | | | | | | | | | | | |
