# Supplementary material for: The older prisoner health and social care assessment and plan (OHSCAP) versus treatment as usual: a randomised controlled trial
Source: BMC Public Health. 2021 Nov 10;21:2061. doi: 10.1186/s12889-021-11965-5 (PMC8579542; doi:10.1186/s12889-021-11965-5)
Supplement: Supplementary file 2 — Additional file 2: Supplementary Material 2. Table 3, Functional health and well-being as measured by the Bristol Activities of Daily Living Scale at 3 months follow-up [file 12889_2021_11965_MOESM2_ESM.docx]

**Supplementary material 2**

| **BADL** |  | **TAU**  **(n = 202)** | **OHSCAP**  **(n = 202)** |
| --- | --- | --- | --- |
| Eating | Needs to be fed | 1 (0) | 0 (0) |
| Dressing | Unable requires total dressing | 0 (0) | 1 (0) |
| Hygiene | Unable needs full assistance | 0 (0) | 1 (0) |
| Bath/shower | Needs full assistance | 0 (0) | 1 (0) |
| Toilet/commode | Taken and given assistance  Incontinent urine or faeces | 0 (0)  1 (0) | 1 (0)  0 (0) |
| Transfers | Gets in chair – needs help out  Totally dependent | 1 (0)  0 (0) | 1 (0)  1 (0) |
| Mobility | Walks with assistance  Uses aids  Unable to walk | 7 (3)  17 (8)  2 (1) | 4 (2)  8 (4)  2 (1) |
| Orientation-time | Unaware but unconcerned | 5 (2) | 3 (1) |
| House (cell) work | Not to required standard  Unable/unwilling to clean | 1 (0) | 1 (0) |

**Table 3: Functional health and well-being as measured by the Bristol Activities of Daily Living Scale at three months follow-up**

None of the participants indicated that they experienced any problems with 10 out of the 19 of the domains of the BADL at three month follow up. These domains were: food; drink; drinking; dressing; teeth; orientation (space); communication; telephone; shopping/canteen and games/hobbies. The domains of the BADL that a minority of participants experienced some difficulties with are detailed in table 4. The most common BADL needs were related to mobility.
